# Wearable Triboelectric/Aluminum Nitride Nano‐Energy‐Nano‐System with Self‐Sustainable Photonic Modulation and Continuous Force Sensing

**DOI:** 10.1002/advs.201903636

**Published:** 2020-06-19

**Authors:** Bowei Dong, Qiongfeng Shi, Tianyiyi He, Shiyang Zhu, Zixuan Zhang, Zhongda Sun, Yiming Ma, Dim‐Lee Kwong, Chengkuo Lee

**Affiliations:** ^1^ Department of Electrical and Computer Engineering National University of Singapore 4 Engineering Drive 3 Singapore 117576 Singapore; ^2^ Center for Intelligent Sensors and MEMS National University of Singapore 5 Engineering Drive 1 Singapore 117608 Singapore; ^3^ NUS Graduate School for Integrative Science and Engineering National University of Singapore 21 Lower Kent Ridge Singapore 119077 Singapore; ^4^ Institute of Microelectronics Agency for Science Technology and Research 2 Fusionopolis Way Singapore 138634 Singapore

**Keywords:** motion monitoring, photonic modulators, triboelectric nanogenerators, wearable textiles

## Abstract

Wearable photonics offer a promising platform to complement the thriving complex wearable electronics system by providing high‐speed data transmission channels and robust optical sensing paths. Regarding the realization of photonic computation and tunable (de)multiplexing functions based on system‐level integration of abundant photonic modulators, it is challenging to reduce the overwhelming power consumption in traditional current‐based silicon photonic modulators. This issue is addressed by integrating voltage‐based aluminum nitride (AlN) modulator and textile triboelectric nanogenerator (T‐TENG) on a wearable platform to form a nano‐energy‐nano‐system (NENS). The T‐TENG transduces the mechanical stimulations into electrical signals based on the coupling of triboelectrification and electrostatic induction. The self‐generated high‐voltage from the T‐TENG is applied to the AlN modulator and boosts its modulation efficiency regardless of AlN's moderate Pockels effect. Complementarily, the AlN modulator's capacitive nature enables the open‐circuit operation mode of T‐TENG, providing the integrated NENS with continuous force sensing capability which is notably uninfluenced by operation speeds. Furthermore, a physical model is proposed to describe the coupled AlN modulator/T‐TENG system. With the enhanced photonic modulation and the open‐circuit operation mode enabled by synergies between the AlN modulator and the T‐TENG, optical Morse code transmission and continuous human motion monitoring are demonstrated for practical wearable applications.

## Graphical abstract

A wearable nano‐energy‐nano‐system featuring the integration of textile triboelectric nanogenerator (T‐TENG) and aluminum nitride photonics is developed. The T‐TENG self‐sustainably transduces mechanical signals into electrical signals that can further modulate optical signals for data transmission. Complementarily, the photonics module offers an optical readout path when the T‐TENG operates under open‐circuit condition to continuously monitor human motions for healthcare applications.

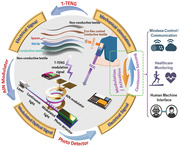

## 1 Introduction

Wearable electronics has rapidly advanced over the past 10 years and nurtured a range of practical applications, including healthcare monitoring,^[^ ^1^, ^2^, ^3^ ^]^ smart displays,^[^ ^4^, ^5^ ^]^ robotics,^[^ ^6^, ^7^, ^8^ ^]^ and energy harvesting.^[^ ^9^, ^10^, ^11^ ^]^ To bridge the conventionally rigid electronics with the conformal human skins, mechanical flexibility and stretchability have been introduced to electronic devices by reducing the film thickness,^[^ ^12^ ^]^ replacing the rigid electronic interconnects by stretchable interconnects while leaving the tiny active devices rigid,^[^ ^13^ ^]^ or using flexible materials in the whole system.^[^ ^14^ ^]^ As demanded by the next‐generation personalized healthcare systems and more accurate multifunctional robotics, a body area sensor network (bodyNET) which hybridizes numerous wearable electronic devices has been proposed.^[^ ^15^ ^]^ With the aid of the development in Internet‐of‐Things (IoT) for real‐time data communication and analysis, instantaneous monitoring and response are envisaged.^[^ ^16^, ^17^, ^18^ ^]^

In the bodyNET and IoT framework, wearable photonics offers a promising platform to complement wearable electronics. On the one hand, the photonic interconnects enable ultra‐fast data transmission as demanded by the (coming) era of IoT.^[^ ^19^, ^20^ ^]^ On the other hand, by using the optical sensing path, the photonic sensors are inert from electromagnetic perturbation which is problematic in the complex bodyNET.^[^ ^21^, ^22^ ^]^ Nonetheless, compared with the rapid development of wearable electronics, the advance in wearable photonics is relatively stagnant. In particular, to realize electronic signal modulation, highly stretchable electronic transistors using polymer materials have been achieved.^[^ ^23^, ^24^, ^25^ ^]^ But the wearable photonic modulator which aims at optical signal modulation for computation functions has not been reported. Despite the successful development of flexible passive waveguides,^[^ ^26^, ^27^, ^28^, ^29^ ^]^ light‐emitting devices including light‐emitting diodes,^[^ ^30^ ^]^ lasers,^[^ ^31^, ^32^ ^]^ and photodetectors,^[^ ^33^, ^34^ ^]^ the lack of flexible photonic modulator is a major bottleneck for the fully integrated wearable photonic system. Besides, conventional photonic modulators are typically realized by silicon photonics and rely on the thermo‐optic effect^[^ ^35^ ^]^ as well as the free carrier dispersion effect.^[^ ^36^ ^]^ With these two types of current‐based modulation mechanisms, the power consumption of photonic modulators increases drastically when there are a few photonic modulators deployed in the bodyNET in order to realize system‐level features, for example, computation and tunable (de)multiplexing. Hence, a novel self‐sustainable photonic modulation mechanism that can significantly reduce the power consumption of wearable photonic systems is strongly desired.

Triboelectric nanogenerator (TENG) based on the coupling between triboelectrification and electrostatic induction has been proven as a safe and self‐sustainable energy source for wearable devices.^[^ ^37^, ^38^, ^39^, ^40^ ^]^ The use of TENG materials and mechanisms paves a new way of making flexible/wearable self‐powered sensors and self‐sustainable electronics systems for healthcare and energy harvesting applications.^[^ ^41^, ^42^, ^43^, ^44^, ^45^, ^46^, ^47^, ^48^, ^49^, ^50^, ^51^, ^52^ ^]^ Because the triboelectric technology shows superior advantages including flexibility/stretch‐ability, high efficiency, versatile operation modes, large output voltage, broad material availability, cost‐effectiveness, and good scalability.^[^ ^53^, ^54^, ^55^ ^]^ Specifically, TENG based on textile materials, called textile TENG (T‐TENG), can be fully integrated with daily worn clothes without causing any additional discomfort. Hence, T‐TENG significantly promotes the flourishing development of next‐generation wearable electronics.^[^ ^56^, ^57^, ^58^, ^59^, ^60^, ^61^, ^62^, ^63^, ^64^ ^]^ The application of TENG in wearable photonics has enabled several self‐sustainable sensing, display, and illumination systems.^[^ ^65^, ^66^, ^67^ ^]^ Yet, regarding photonic modulation, the low‐current feature of TENG is incompatible with the conventional silicon photonic modulators which require a large current supply. Alternatively, aluminum nitride (AlN) photonic modulators based on the Pockels effect require low‐current but high‐voltage for modulation.^[^ ^68^, ^69^ ^]^ The recent report on flexible AlN photonics also shows its potential for wearable photonics applications.^[^ ^70^ ^]^ Moreover, the synergy between triboelectric technology and AlN photonics can offer extra benefits to both sides. The high‐voltage from TENG can be applied to the AlN modulator with negligible degradation and effectively enhance the modulation efficiency through bypassing the limited tuning efficiency restricted by AlN's moderate Pockels effect. Complementarily, the capacitor nature of AlN modulator and the optical transmission capability of photonic systems could be another possible solution to continuously monitor the TENG output in a compact and easy‐to‐implement manner other than the conventional open‐circuit voltage/charge approach which relies on bulky and complicated external electrical circuits.

Here, we report the integration of AlN modulator and T‐TENG on a wearable platform to construct the wearable nano‐energy‐nano‐system (NENS) for self‐sustainable photonic modulation. The wearable NENS is simultaneously equipped with continuous force sensing function. The self‐generated high‐voltage from T‐TENG enhances the modulation depth of the AlN modulator through the Pockels effect. Based on the photonic modulation function, optical Morse code transmission is demonstrated for encoded data transmission. On top of the enhanced photonic modulation in AlN modulator, the capacitive nature of AlN modulator benefits T‐TENG by enabling its open‐circuit operation mode, which is subsequently utilized for continuous force sensing. Notably, the continuous force sensing is uninfluenced by different impact force speeds on T‐TENG. A physical model that describes the coupled AlN modulator/T‐TENG system is proposed to obtain a semi‐analytical formula for deriving a calibration curve that provides a one‐to‐one relation between the optical transmission and the impact force magnitude. Continuous force sensing, where the sensing data is transmitted instantaneously and wirelessly through the optical signals, is achieved for continuous human motion monitoring. Under the auspices of T‐TENG, the generated high voltage output, the excellent optical tuning feature, and the open‐circuit operation mode of the wearable NENS pave the way to future self‐sustainable wearable tunable photonics for communication, healthcare monitoring, and human‐machine interface applications.

## 2 Concept of the Wearable Triboelectric/AlN NENS

**Figure 1:**
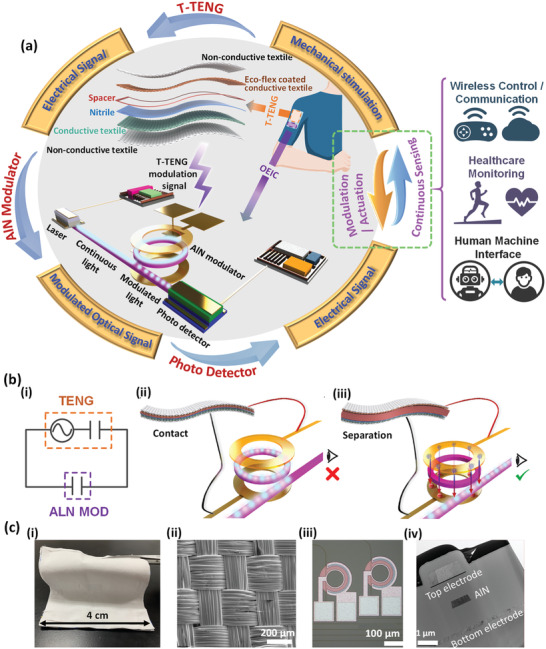
Proposed wearable triboelectric/aluminum nitride (AlN) nano‐energy‐nano‐system (NENS) featuring the integration of AlN modulator and textile triboelectric nanogenerator (T‐TENG) on a wearable platform. a) Wearable NENS comprised of a tiny rigid AlN photonic module (purple) and a T‐TENG module serving as a high voltage source (orange). Mechanical, electrical, and optical signals can be transduced in the system to achieve wide photonic modulation and continuous force sensing. The details of the AlN photonics module and the exploded view of the T‐TENG are presented, as well as the potential applications of the system. b) Equivalent circuit model diagram (i) and fundamental working principle of the integrated system (ii and iii). c) T‐TENG optical image (i); conductive textile SEM image (ii); AlN modulator optical (iii); and TEM image (iv).

The integration of AlN modulator and wearable T‐TENG is inevitable to take advantage of both devices and enable their synergy. The proposed wearable NENS with self‐sustainable photonic modulation and continuous force sensing functions features a wearable platform comprised of a tiny rigid AlN photonic module (purple) and a T‐TENG module (orange) as shown in **Figure** **1**a. As illustrated in the outer signal flow circle, fundamentally the self‐sustainable photonic modulation is realized by using the T‐TENG as a power supply. The T‐TENG transduces mechanical stimulations to electrical signals based on the coupling of triboelectrification and electrostatic induction. The self‐generated electrical signals are then applied to the AlN modulator to generate modulated optical signals, which are routed to photodetectors and converted to electrical readouts. Complementarily, the continuous force sensing is realized by using the AlN modulator for sensing signal readout. Such a readout scheme decouples the sensing path and the signal readout path so that high optical readout signals can be received even when the electrical sensing circuit is operated with low output current. The detailed device configurations of both the AlN modulator and the T‐TENG are shown inside the signal flow circle. The AlN modulator is composed of an AlN microring resonator (MRR) sandwiched by a pair of top and bottom electrodes to leverage AlN's *r*_13_ electro‐optic (EO) coefficient. The electrodes are connected to the T‐TENG output. As for the T‐TENG module, flexible Ecoflex and nitrile layers on conductive textiles are adopted as the negative and positive friction surface respectively. Upon physical contact, opposite charges with equal quantity are generated on the two surfaces due to their different electron affinities. Upon separation of the two charged surfaces, the built‐up electric potential difference will induce an output voltage in the external circuit. A thin spacer is sandwiched between two functional layers for separation. The entire structure is encapsulated by two additional pieces of non‐conductive textiles. Figure 1b explains the basic working mechanism of the system. In the equivalent circuit model diagram (Figure 1b(i)), the T‐TENG can be considered as a serial connection of an alternating current (AC) voltage source and a capacitor, while the AlN modulator acts as a parallel plate capacitor. The AlN MRR is initially working on resonance where the optical transmission is zero at the output (Figure 1b(ii)). A zero‐bias is applied to the AlN MRR when the T‐TENG is in the contact mode since opposite charges are neutralized at the contact interface. Contrarily, a high‐voltage is applied to the AlN MRR when the T‐TENG is in the separation mode due to the electrostatic induction. The generated strong electric field (E‐field) alters AlN's refractive index through the Pockels effect and consequently changes the resonant condition. The AlN MRR then operates in the off‐resonance condition, and a measurable optical transmission is received at the output (Figure 1b(iii)). The intensity of the optical transmission depends on the voltage from the T‐TENG. Figure 1c shows the 4 × 4 cm^2^ wearable T‐TENG optical image (Figure 1c(i)), the conductive textile scanning electron microscope (SEM) image (Figure 1c(ii)), the AlN modulator optical image (Figure 1c(iii)) as well as its tunneling electron microscope (TEM) image (Figure 1c(iv)). The resistance and capacitance of the AlN modulator are 2.1 GΩ and 2.1 nF respectively.

## 3 Characteristics of the T‐TENG and the AlN Modulator

**Figure 2:**
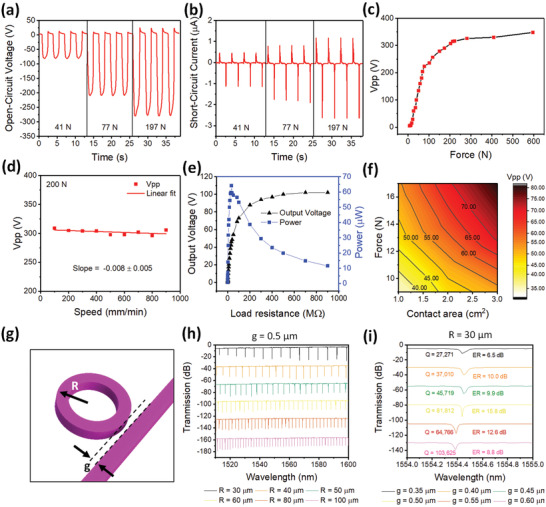
Characteristics of the T‐TENG and the AlN MRR. a) Open‐circuit voltage under different applied periodic forces. b) Short‐circuit current under different applied periodic forces. c) Dependence of open‐circuit *V*_pp_ on the impact force magnitude. d) Load cell speed dependence of open‐circuit *V*_pp_ under constant impact force magnitude at 200 N. e) Output voltage and output power of the T‐TENG module at different load resistances. f) Contour map showing the dependence of open‐circuit *V*_pp_ on impact force magnitude and contact area. g) Schematic of the AlN MRR. h) Resonance characteristics of AlN MRR with fixed *g* but a varying *R*. i) Resonance characteristics of AlN MRR with fixed *R* but a varying *g*.

The fabricated T‐TENG should generate sufficient voltage and power to enable enhanced photonic modulation and even sustain the entire wearable NENS. First, the basic characterization of the T‐TENG is conducted using a force gauge testing system that provides impact forces with controllable magnitudes by a load cell with varying speeds. **Figure** **2**a shows the typical open‐circuit voltage waveforms of the T‐TENG under different impact force magnitudes of 41, 77, and 197 N, respectively (at 900 mm min^−1^ load cell speed). A clear improvement of the open‐circuit voltage along with an increasing impact force magnitude can be observed. Accordingly, the short‐circuit current of the T‐TENG under the same impact force magnitudes is presented in Figure 2b, exhibiting the same improvement trend. The detailed relationship of the open‐circuit voltage (peak to peak value *V*_pp_) and the impact force magnitude at 900 mm min^−1^ load cell speed is obtained from Figures S1 and S2, Supporting Information, then plotted in Figure 2c. It is observed that *V*_pp_ increases rapidly and linearly in the low impact force magnitude range (<200 N), and then gradually saturates in the higher impact force magnitude range (>200 N). A maximum *V*_pp_ of 350 V can be achieved under 600 N force. The *V*_pp_ increment is associated with the larger amount of charges generated due to the stronger material surface interaction under a higher impact force magnitude. Besides the impact force magnitude, the load cell speed is a critical parameter when the T‐TENG is used in practical applications. Thus, the relationship between *V*_pp_ and the load cell speed (at 200 N force) is also investigated and shown in Figure 2d whose data is extracted from the waveforms presented in Figure S3, Supporting Information. As expected, a highly stable *V*_pp_ is observed at various speeds as a result of T‐TENG's open‐circuit operation mode, demonstrating that the *V*_pp_ of T‐TENG is only determined by the impact force magnitude but independent of the load cell speed. To examine the power generation capability of the T‐TENG, *V*_pp_ from different resistor loads when they are connected to the T‐TENG (at 200 N force) are measured. The corresponding output power is then calculated by *P* = *V* ^2^/*R* and plotted in Figure 2e. A maximum output power of 64 µW can be achieved when the connected resistor load is 33 MΩ. Toward practical wearable applications, the output performance of the T‐TENG is also measured by finger tapping in the scenarios of using one, two and three fingers. The measuring approach is illustrated in Figure S4, Supporting Information. The resultant *V*_pp_ under various impact force magnitudes and contact areas (different number of fingers) is presented in Figure 2f, showing an apparent increment of *V*_pp_ with both larger impact force magnitudes and contact areas. Therefore, output voltage with adjustable magnitudes can be easily achieved by controllable human tapping in order to realize targeted photonic modulation applications.

A high‐performance AlN modulator is required for high‐speed optical transmission, on‐chip computation, and effective tuning. In order to systematically characterize the AlN modulators, an array of AlN modulators is fabricated. The characteristic of the AlN modulator is fundamentally determined by the ring radius (*R*) and coupling gaps (*g*) of the AlN MRR (Figure 2g). According to the MRR resonant condition, the resonant wavelength *λ*_R_ is solely determined by *R*. Thus, by varying *R*, *λ*_R_ can be designed to operate at specific wavelengths. The free spectral range (FSR) associated with the spacing between different *λ*_R_s will change with *R* as well. The spectra of the AlN MRRs with fixed *g* = 0.5 µm and different *Rs* are plotted in Figure 2h while the corresponding measured *λ*_R_ around 1555 nm and FSR are presented in Note S3, Supporting Information. *λ*_R_ is positively related to *R* while the FSR decreases with an increasing *R*. The resonant lineshape can be effectively tailored by a varying *g*. As shown in Figure 2i, when *R* is fixed at 30 µm, by increasing *g* from 0.35 to 0.60 µm in step of 0.05 µm, *λ*_R_ remains constant at 1554.429 ± 0.033 nm. The expected *λ*_R_ is insensitive to the change in *g*. However, Figure 2i also indicates that the quality factor (Q factor) and the extinction ratio (ER) are strongly dependent on the coupling gap. The Q factor can be superior to 100 K at the under‐coupling condition (*g* = 0.60 µm) while the ER can reach a maximum of 15.8 dB near the critical‐coupling conditions (*g* = 0.50 µm).

**Figure 3:**
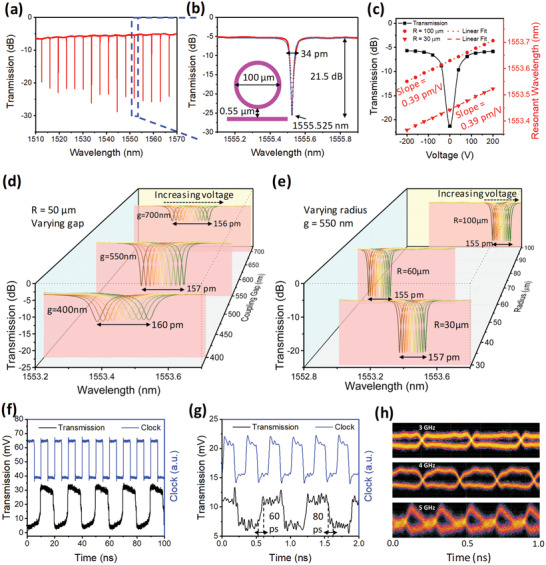
Characteristics of the AlN modulator under DC and AC bias. a) Transmission spectrum of the AlN MRR (*R* = 50 µm, *g* = 0.55 µm) in 1510 to 1570 nm. b) Zoom‐in spectrum to the resonant wavelength at 1555.525 nm. c) DC tuning of the optical transmission in the *R* = 50 µm, *g* = 0.55 µm AlN MRR and the resonant wavelengths in other two AlN MRRs (*R* = 30 µm, *g* = 0.55 µm and *R* = 100 µm, *g* = 0.55 µm). d) Coupling gap dependence of the DC tuning efficiency. e) Radius dependence of the DC tuning efficiency. f) AC modulation of the AlN modulator at 100 MHz. g) AC modulation of the AlN modulator at 3 GHz. h) Temporally accumulated modulation signals at 3, 4, and 5 GHz.

An AlN MRR with *R* = 50 µm and *g* = 0.55 µm is first used to characterize the modulation performance of the AlN modulator. The optical transmission spectrum of the AlN MRR around the telecommunication C‐Band is presented in **Figure** **3**a. An average insertion loss of 5.73 dB and a FSR of 3.612 nm is demonstrated. Figure 3b zooms into the resonant wavelength at 1555.525 nm, revealing a 3‐dB bandwidth of 34 pm which corresponds to a Q factor of 45750. An ER of 21.5 dB is accompanied. The resonant wavelength at 1555.525 nm is then chosen for further modulation investigation because it is close to the 1550 nm telecommunication wavelength. Besides, it possesses satisfactory Q factor and ER that benefit the modulation efficiency. Next, we investigate the performance of the AlN modulator under different applied biases. The optical transmission of the *R* = 50 µm, *g* = 0.55 µm AlN MRR at different applied biases is shown in Figure 3c. The tuning efficiency is high in the low voltage range and decreases in the high voltage range due to the Lorentzian lineshape of the corresponding resonance. Figure 3c simultaneous presents the direct current (DC) tuning of the resonant wavelengths in other two AlN MRRs (*R* = 30 µm, *g* = 0.55 µm and *R* = 100 µm, *g* = 0.55 µm). The resonant wavelength can be continuously tuned in the range from 1553.366 to 1553.523 nm and 1553.551 to 1553.706 nm respectively when a −200 to 200 V DC bias is applied. A linear relationship between *λ*_R_ and the applied voltage is observed with a tuning efficiency of 0.39 pm V^−1^. To further understand the influence of the undesired but inevitable fabrication variations on the tuning efficiency, we investigate the dependence of tuning efficiency on *R* and *g*. As shown in Figure 3d, in AlN modulators with *R* = 50 µm but different *g*s, *λ*_R_ shifts 157.67 ± 2.08 pm when a 400 V bias difference is introduced. As for AlN modulators with *g* = 0.55 µm but different *R*s, Figure 3e shows a *λ*_R_ shift (Δ*λ*_R_) of 155.67 ± 1.15 pm under a 400 V bias difference. The results from Figure 3d,e together indicate the tuning efficiency of AlN modulators is robust and unaffected by the varying *R*s and *g*s. This inert response of tuning efficiency to the AlN modulator geometry variation is further confirmed through a theoretical analysis (Note S4, Supporting Information). Furthermore, to confirm the high‐quality of the deposited AlN thin film, the *r*_13_ EO coefficient of AlN is extracted by simulation and calculation (Note S5, Supporting Information). AlN's *r*_13_ EO coefficient of 1.4 pm V^−1^ is obtained, which agrees well with the reported result.^[^ ^69^ ^]^

The high‐speed modulation capability of the AlN modulator is investigated by applying square waves with 10 *V*_pp_ and + 5 V bias. To achieve the highest ER, the device is working at 1555.525 nm which is the resonant wavelength that results in zero transmission without applying bias. As shown in Figure 3f, the modulation is efficient at 100 MHz modulation frequency. There is a negligible phase delay between the input radio frequency (RF) signal and the modulated optical signal. The lowest and highest optical transmission is 3.7 and 32.4 mV respectively, corresponding to an ER of 9.4 dB. Figure 3g plots the modulation results at 3 GHz. Since the RF source has a maximum speed of 12.5 GHz, some RF signal distortions away from a square waveform are observed at 3 GHz in the clock signal. The AlN modulator still carries the input RF signal efficiently despite some small phase delays and a reduced ER of 2.12 dB. The rise time *τ*_r_ and fall time *τ*_f_ (defined by 10% and 90% of the step height) is 60 and 80 ps respectively. A rough estimation of the cut‐off frequency can be calculated as 2 GHz by

(1) $$\;f\approx \frac{1}{2\pi \times \max\left(\tau_{r},\tau_{f}\right)}$$

The cut‐off frequency is further verified by the optical transmission signal accumulated temporally as shown in Figure 3h. The light is effectively modulated by the 3 GHz RF input. At 4 GHz, severe waveform distortion happens. And the AlN modulator fails to carry the RF signal at 5 GHz. In order to figure out the limiting factor of the maximum 3 GHz modulation speed, we estimate the photon lifetime *τ*_p_ in the MRR according to

(2) $$Q=2\pi \times f\times \tau_{p}$$

where *Q* is the Q factor of the ring resonator, *f* is the light frequency. *τ*_p_ of around 40 ps is estimated, which is close to the measured rise time and fall time. Thus, we believe the limiting factor in the AlN modulator is the long photon lifetime. In order to increase the modulation speed, a modulator with a lower Q factor can be designed to reduce *τ*_p_. With a moderate Q factor of 4000, the theoretical modulation speed limited by the photon lifetime can reach 20 GHz. Meanwhile, the top and bottom electrodes design also needs careful consideration to ensure low RC delay.

## 4 Operation Principle of the Wearable Triboelectric/AlN NENS

**Figure 4:**
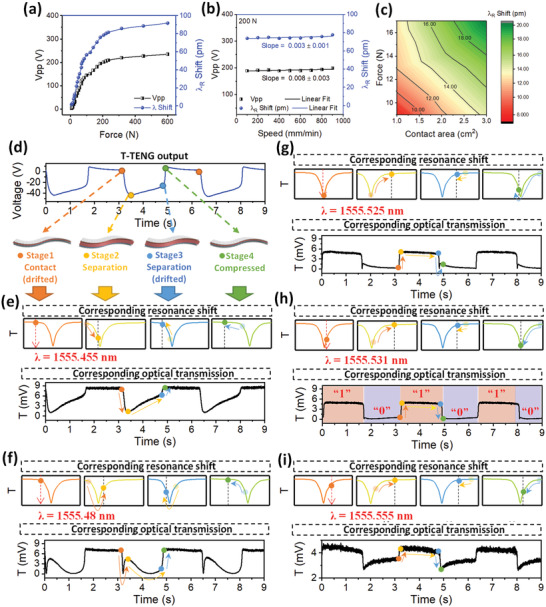
Operation Principle of the wearable triboelectric/AlN NENS for self‐sustainable photonic modulation. a) Impact force magnitude dependence of *V*_pp_ applied on the AlN MRR and the corresponding resonant wavelength shift. b) Load cell speed dependence of *V*_pp_ applied to the AlN MRR and the corresponding resonant wavelength shift. c) Contour map showing the dependence of resonant wavelength shift on impact force magnitude and contact area. d) T‐TENG output voltage waveform generated by periodic motion of the force gauge and the characteristic T‐TENG stages in terms of contact/separation mode. e–i) Resonance wavelength shift induced by T‐TENG voltage output and the corresponding optical transmission waveform at the operation wavelength of 1555.455 nm (e), 1555.48 nm (f), 1555.525 nm (g), 1555.531 nm (h), 1555.555 nm (i). The best self‐sustainable photonic modulation is achieved in (h).

In the integrated wearable NENS, the two electrodes from the T‐TENG are connected to the top and bottom electrodes that sandwich the AlN MRR, forming the electrical‐photonic tuning system. It is significant to ensure that the high‐performance of the AlN modulator and the T‐TENG is maintained after integration. The output characteristics of the T‐TENG when it is integrated with the AlN modulator are first studied. As shown in **Figure** **4**a, the *V*_pp_ increases rapidly with impact force magnitude in the low impact force magnitude range and saturates at 235 V gradually in the high impact force magnitude range. This relationship has the same trend as the open‐circuit voltage from the standalone T‐TENG in Figure 2c, with the only difference in the absolute voltage magnitude. The reduction of voltage magnitude is mainly caused by the Bayonet Neill–Concelman (BNC) cables that are used to connect the AlN modulator and the T‐TENG for characterization (Note S6, Supporting Information). In practical applications, the T‐TENG will be directly connected to the AlN modulator without BNC cables and introduces almost no voltage reduction due to the minuscule capacitance of the AlN modulator. Based on the output characteristic, the corresponding Δ*λ*_R_ can be obtained by the 0.39 pm V^−1^ sensitivity because the electrical signal from the T‐TENG is far slower than the intrinsic response time of the AlN modulator. Around 100 pm Δ*λ*_R_ can be achieved in the integrated system. Next, the dependence of *V*_pp_ and corresponding Δ*λ*_R_ on the load cell speed (at 200 N impact force magnitude) is also investigated and presented in Figure 4b. The *V*_pp_ and Δ*λ*_R_ are both unaffected by the load cell speed and only determined by the impact force magnitude. This independence of *V*_pp_ on the load cell speed further confirms that the T‐TENG is working in the open‐circuit condition due to the capacitive nature of the AlN modulator. Testing of the T‐TENG as a human‐machine interface is implemented. With finger tappings as the triggering, the resultant Δ*λ*_R_ shows notable increment with impact force magnitudes and contact areas. As for the AlN modulator, its optical characteristics are completely maintained since the electrical connection does not affect the optical path.

The detailed self‐sustainable photonic modulation mechanism and phenomenon including the open‐circuit voltage from the T‐TENG and the transmission spectrum from the AlN modulator is presented in Figure 4d–i. The open‐circuit voltage from the T‐TENG has a periodic alternating waveform that is similar to a square‐wave (Figure 4d). It is generated by the force gauge periodic motion. The deviation from a square‐wave is caused by the dissipation of charges to the humid environment (60% relative humidity at room temperature). Another possibility of charge dissipation to the electrical circuit is excluded, because the square‐wave is still non‐ideal when the T‐TENG only works in the open‐circuit condition where charges do not flow. We examine the detailed T‐TENG's output characteristics (Figure 4d) and the corresponding Δ*λ*_R_ (Figure 4e) in one cycle. At Stage 1, the load cell is in full contact with the T‐TENG with the pre‐set impact force magnitude (contact mode of the T‐TENG), and the respective open‐circuit voltage is zero. Then from Stage 1 to Stage 2, the load cell is moving up to the zero position and the two triboelectric layers are gradually separated from each other (separation mode of the T‐TENG) due to the device restoring force. An electric potential is rapidly built up with the separation, inducing a significant increment in the open‐circuit voltage. The negative output is determined by the direction of electrode connection. The large negative voltage blueshifts *λ*_R_. At Stage 2, the load cell is back to the zero position (separation mode of the T‐TENG) and the open‐circuit voltage reaches the negative maximum. *λ*_R_ at this stage is at the leftmost position (Figure 4e). Next, from Stage 2 to Stage 3, the slow decrement of open‐circuit voltage is due to the system charge dissipation when the load cell is held still at zero position. Correspondingly, *λ*_R_ slowly redshifts. Then from Stage 3 to Stage 4, the load cell is moving down to contact with the T‐TENG again (contact mode of the T‐TENG). Thus, the open‐circuit voltage decreases toward zero rapidly during this period. Accordingly, a rapid redshift of *λ*_R_ is observed. It is worth to note that the open‐circuit voltage at Stage 4 is above zero with maximum impact force magnitude, due to the drifting of the open‐circuit voltage. Finally, the open‐circuit voltage of T‐TENG is drifted back to zero (Stage 1) and the next cycle begins.

The optical transmission waveform is strongly dependent on the operation wavelength. Therefore, the optimal operation wavelength should be identified at the first use of the system to ensure the best self‐sustainable photonic modulation performance. In Figure 4e, when the system is working at 1555.455 nm while *λ*_R_ is constant at 1555.525 nm with zero bias, the optical transmission waveform almost reproduces the T‐TENG output voltage waveform. When the operation wavelength is 1555.48 nm which is closer to *λ*_R_, a different waveform featuring a sharp though from Stage 1 to Stage 2 is presented in Figure 4f. As shown in the corresponding resonance shift, from Stage 1 to Stage 4, *λ*_R_ consecutively blue shifts strongly, redshifts slightly, and redshifts strongly. In each subfigure, the observed transmission from the previous stage is also marked in a colored transparent circle. At Stage 1, the optical transmission is high since the operation wavelength is to the left of *λ*_R_. From Stage 1 to Stage 2, due to the large blueshift, *λ*_R_ approaches, coincides with, and further blueshifts to the left side of the working wavelength, resulting in a sharp valley in the transmission. From Stage 2 to Stage 3, *λ*_R_ slowly redshifts, approaches, coincides with, and further redshifts to the right side of the working wavelength, inducing a slow transaction of decrement and increment in transmission. Then from Stage 3 to Stage 4, a significant increment in transmission can be observed due to the rapid redshift of *λ*_R_. Similarly, from Stage 4 to Stage 1, the open‐circuit voltage gradually decreases back to zero when the load cell maintains in full contact with the T‐TENG. Due to the small decrement of voltage, *λ*_R_ only slightly blueshifts, maintaining the high transmission level. At other working wavelengths, the operation principle is similar. Figure 4g shows that when the operation wavelength is exactly at *λ*_R_, the optical transmission waveform features a sharp though from Stage 3 to Stage 4. The best self‐sustainable photonic modulation can be achieved when the operation wavelength is at 1555.531 nm, slightly longer than *λ*_R_, as illustrated in Figure 4h. A square optical transmission waveform is obtained without the presence of any though. Thus we can easily define “1” and “0” to realize binary operations. When the operation wavelength further moves to a longer wavelength at 1555.555 nm, the photonic modulation depth becomes shallow while the optical transmission waveform is the reverse of the initial T‐TENG output voltage waveform. Besides the five characteristic optical transmission waveforms shown in Figure 4e–i, a full set of 21 optical transmission waveforms when the system operates at 21 different wavelengths is presented in Note S7, Supporting Information. These distinct optical transmission waveforms have the potential to serve as symbols for communication and human‐machine interface applications.

**Figure 5:**
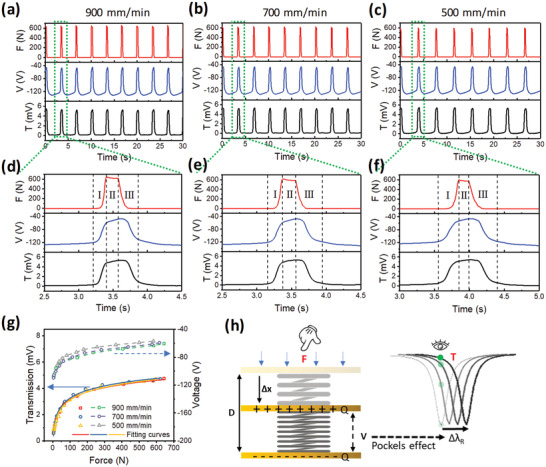
Operation Principle of the Wearable Triboelectric/AlN NENS for continuous force sensing. a–c) The impact force magnitude, the resultant applied voltage on the AlN modulator, together with the optical transmission spectrum at different load cell speeds of, 900 (a), 700 (b), and 500 mm min^−1^ (c). d–f) Zoom‐in of the spectrum in (a–c) to a complete operation cycle, 900 (d), 700 (e), and 500 mm min^−1^ (f). g) The relationship of the resultant optical transmission, the T‐TENG output voltage, and the impact force magnitude at different load cell speeds. The fitted curves are calibration curves for reading the impact force magnitude from the optical transmission. h) Proposed physical model that describes the integrated system. The T‐TENG is described by a parallel plate capacitor where the two plates are connected by a spring. The AlN MRR is described by a resonator with a Lorentzian resonant lineshape.

As for continuous force sensing, conventionally it is only applicable when the TENG operates in the open‐circuit mode but not the closed‐circuit mode. Because in the closed‐circuit mode, the charging/discharging process through the external circuit will screen the generated surface charges, during which the two output pulses in an operation cycle cannot fully reflect the continuous force information. Therefore, bulky and complicated external electrical circuits are required to activate TENG's open‐circuit operation mode. Thanks to the capacitive nature of the AlN modulator, a superior advantage of the integrated wearable NENS is the capability of enabling T‐TENG to work under the open‐circuit mode in a compact and easy‐to‐implement manner. While the T‐TENG serves for continuous force sensing in the integrated system, the photonic module helps with transmitting the open‐circuit sensing signal out in‐situ through optical signal, which can be detected by a photodetector circuit. In the experiment, the wearable NENS is working similarly to Figure 4e where the optical transmission spectrum replicates the waveform of the T‐TENG modulating signal. The detail waveforms of the impact force, the resulting induced voltage on the AlN modulator, and the optical transmission spectrum are shown in **Figure** **5**a–c, with different load cell speeds of 900, 700, and 500 mm min^−1^. Figure 5d to Figure 5f present the zoom‐in waveforms in a complete cycle, corresponding to the three load cell speeds respectively. As indicated by the four dash lines in the graph, a cycle can be divided into three stages from left to right with respect to the force status applied on the T‐TENG. At Stage I, initially, the load cell is approaching and gradually touches the T‐TENG. Because of T‐TENG's membrane/air‐gap/substrate structure, the distance between the two friction surfaces decreases significantly with a small impact force magnitude, leading to an abrupt change in the T‐TENG output voltage. As a result, the optical transmission from the AlN modulator drastically increases, following the same trend as the T‐TENG output voltage visually. As the load cell further approaches and compresses the T‐TENG, the two friction surfaces are in full contact. The impact force magnitude boosts to the pre‐set value. But the T‐TENG output voltage and the corresponding optical transmission only vary slowly since the distance between the two friction surfaces cannot be changed effectively. At Stage II, the load cell is in full contact with the T‐TENG with the pre‐set impact force magnitude; and the induced voltage is maintained at a low level. The induced voltage is not zero at this stage due to the open‐circuit voltage shift arisen from the measurement instrument, while the small decrement of the induced voltage toward zero can be attributed to the slow charge dissipation of the system. A similar trend can also be found from the optical transmission of the AlN modulator. Then at Stage III, the load cell is removed from and gradually releases the T‐TENG. Initially, the impact force magnitude decreases significantly from the full contact state to the critical contact state where the two friction surfaces are still in contact with each other, but the impact force magnitude is at a very low level. Thus, during this period, the induced voltage on the AlN modulator remains high. When the load cell is further removed, the two friction surfaces start to separate from each other; and the induced voltage on the AlN modulator gradually increases accordingly. Similarly, the same trend can also be found in the optical transmission waveform. Through analyzing the measurement results from different load cell speeds, the absolute value of the induced voltage as well as the optical transmission in the AlN modulator can reach and maintain a monotonous value that corresponds solely to the absolute magnitude of the impact force but not affected by the load cell speeds. At faster load cell speeds, both the induced voltage and the optical transmission exhibit a higher increment or decrement rate, but the maximum values still maintain the same. It means that the optical transmission of the AlN modulator can follow the exact trend of the applied force on the T‐TENG, providing an advanced and easy‐to‐implement approach for continuous force sensing compared to the open‐circuit voltage measurement.

The clear relationship of the resultant optical transmission, the T‐TENG output voltage, and the impact force magnitude at different load cell speeds is plotted in Figure 5g whose data is extracted from Stage I in Figure 5d–f. The fitted curves are calibration curves for reading the impact force magnitude from the optical transmission quantitatively and accurately when the load cell presses the NENS (Stage I). As a fundamental requirement, the calibration curve should have a one‐to‐one relationship. The monotonically increasing optical transmission with the increasing impact force magnitude suffices to fulfill the calibration curve requirement. A theoretical analysis is implemented to provide an analytical formula that can depict the full relationship between the impact force magnitude and the optical transmission, instead of only the measured discrete points. A physical model is proposed to describe the integrated system (Figure 5h). The T‐TENG is described by a parallel plate capacitor where the two plates with constant positive charges and negative charges are connected by a spring dominated by the stress‐strain relation. The AlN MRR is described by a resonator with a Lorentzian resonant lineshape where the resonant wavelength is determined by the E‐field across the parallel plate capacitor. The force on spring (*F*) determines the parallel plate capacitor's voltage (*V*) and subsequently causes Δ*λ*_R_ through the Pockels effect. The resultant optical transmission (*T*) at the specific wavelength is tuned continuously and follows the Lorentzian lineshape. After such formulation and with aid of the pre‐determined Lorentzian resonant lineshape shown in Figure 3b, the relation between the optical transmission and the impact force magnitude can be expressed as

(3) $$\begin{array}{ccc} \;T & = & \frac{2B}{\pi }\times 3.4\times 10^{-2}\\ & & \times \left[-\frac{1}{1.0372\times 10^{-2}}+\frac{1}{\begin{array}{c} 1.156\times 10^{-3}+6.4\times 10^{-7}\times \\ {\left[\frac{120}{D}\times \left(D-{\left(\frac{F}{K}\right)}^{\frac{1}{N}}\right)\right]}^{2} \end{array}}\right] \end{array}$$

where *T* is the optical transmission and *F* is the impact force magnitude. *D, K, N, B* are fitting parameters where *D* is the separation between two plates without applying force, *K* and *N* are two coefficients in the stress‐strain relationship, *B* is a mathematical fitting parameter without physical meaning. The detailed derivation is presented in Note S8, Supporting Information. The data in Figure 5g is fitted well by the derived expression. More significantly, the fitted calibration curve is independent of the speed of the impact force. This benefits the continuous force sensing in practical applications.

However, as shown in Figure 5g, in the current NENS design, the force sensing is only sensitive up to around 200 N impact force magnitude, after which the optical transmission only changes slightly with the increasing impact force magnitude. The 200 N saturation point corresponds to the T‐TENG state when the two friction surfaces are in full contact. In order to extend the sensitive range, a spacer material with a larger strength coefficient can be used so that a larger force is required to change the distance between the two friction surfaces and reach the full‐contact state.

## 5 Diversified Applications of the Wearable Triboelectric/AlN NENS

**Figure 6:**
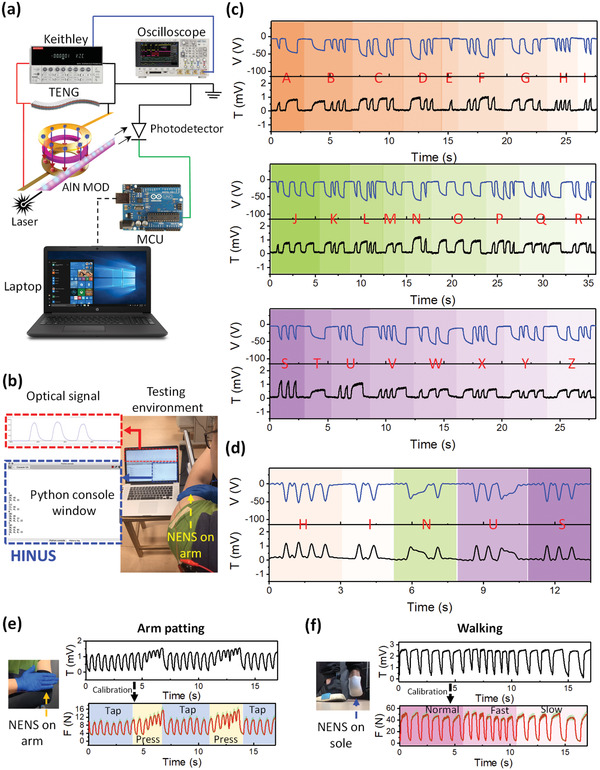
Diversified Applications of the Wearable Triboelectric/AlN NENS. a–d) Optical Morse code transmission, characterization setup (a), interface of the home‐built optical Morse code reader software (b), the 26 distinguished alphabets transmitted by the optical Morse code (c), optical transmission of the information “HINUS” using the wearable NENS (d). e,f) Continuous human motion monitoring. The optical transmission spectrum (top) and the corresponding translated impact force magnitude (bottom) resulted from, arm patting (e), and walking (f). The calibration relies on the calibration curve in Figure 5g.

One of the major applications of wearable photonics is data transmission. Based on the self‐sustainable photonic modulation and tuning function, the wearable NENS is first leveraged for optical Morse code transmission. **Figure** **6**a shows the schematic of the corresponding measurement system. The optical signals are converted to voltage signals by the photodetector and subsequently directed to a microcontroller unit (MCU) that serves as the interconnect between the integrated system and the computer. The Keithley electrometer together with the oscilloscope help to monitor the real‐time voltage output from the T‐TENG. Once the processed signal is received by the computer, the home‐built software shown in Figure 6b can translate the detected signal into the pre‐defined alphabets whose array could convey the desired messages. Figure 6c demonstrates the transmission of all the 26 alphabets. And a message array conveying “HINUS” is shown in Figure 6d. A video showing the dynamic Morse code transmission of “HINUS” is presented in Video S1, Supporting Information. In principle, all alphabets combinations are feasible to transmit arbitrary meaningful messages. In the optical Morse code demonstration, long and short optical pulses are used to define the “bar”s and “dot”s respectively for communication. Furthermore, the self‐sustainable photonic modulation function can be used to generate arrays of “0”s and “1”s. The arrays could serve as symbols for machine control,^[^ ^71^ ^]^ where the NENS is used as a human‐machine interface.

Another key application of wearable photonics is sensing. At the same time, triboelectric technology is also widely adopted in various sensing applications. With the help of an integrated AlN modulator in the wearable NENS, continuous force sensing can be achieved by taking advantage of merits from both sides. Here, we demonstrate the continuous human motion monitoring using the wearable NENS. Two monitoring scenarios of continuous force when the textile wearable system is attached to the human arm and sole are investigated. For the device attached on arm (Figure 6e top panel), cyclic finger tapping is applied on the device during the first 5 s. Then the fingers remain in contact with the device and press the device periodically for around 2 s. The same motion pattern is repeated starting from the cyclic finger tapping. For the device attached on sole (Figure 6f top panel), three walking patterns are subsequently applied on the device, that is, normal walking, fast walking, and slow walking. Videos for these two monitoring scenarios can be found in Videos S2 and S3, Supporting Information. The corresponding force spectra (Figure 6e,f bottom panel) are derived by the pre‐determined calibration curve (Figure 5g), and the shaped green area represents the corresponding uncertainties caused by the variation in the impact force speed. The derived force spectrum from optical transmission replicates the actual impact force magnitude spectrum in a real‐time manner, showing great potentials of the integrated system in practical applications for continuous force/pressure monitoring.

Although directly connecting the TENG output to the MCU is a convenient connection approach to use the TENG as a self‐sustainable human‐machine interface. However, while the TENG serves as a self‐sustainable triggering signal generator in the circuits, a load resistance in the MCU reads the TENG signal. Consequently, the TENG is working in the closed‐circuit condition. One major drawback when the TENG works in the closed‐circuit condition is that the charging/discharging process through the external circuit will screen the generated surface charges, during which the two output pulses in an operation cycle cannot fully reflect continuous information. The AlN modulator is electrically capacitor‐like. Thus, when connecting to an AlN modulator, the TENG is working in the open‐circuit condition. Although the current does not flow, the optical signal carries the TENG triggering signal information which is converted to an electrical signal by a photodetector. And finally, the photodetector signal carrying the same information as the open‐circuit TENG signal is read by the MCU which is not achievable if the TENG is directly connected to the MCU. In order to clearly illustrate the advantages of using the AlN photonic modulator in the T‐TENG + modulator + MCU system as compared to the simple T‐TENG + MCU system, we demonstrate a direct comparison between the signal generated in the TENG + photonics + MCU system and the signal generated in the TENG + MCU system for the Morse code transmission and continuous human motion monitoring applications. The results are shown in Note S9, Supporting Information.

## 6 Conclusions

In summary, we have developed a wearable triboelectric/aluminum nitride NENS with self‐sustainable photonic modulation and continuous force sensing functions. While the previous studies focus on wearable optical illumination and optical detection, our work aims at optical modulation and addresses the power consumption issue in modulator systems by integrating voltage‐based AlN modulator and T‐TENG power source. The synergy between AlN modulator and T‐TENG brings two major advantages to the integrated system. On the one hand, despite AlN's moderate Pockels effect, the enhanced modulation is achieved in AlN modulators enabled by T‐TENG's high voltage output. On the other hand, T‐TENG's open‐circuit operation mode can be facilitated by AlN modulator's capacitive nature and consequently provides a compact and easy‐to‐implement system for continuous sensing. The characterization of individual AlN modulator and T‐TENG shows superior device performance respectively in terms of a high‐quality resonance lineshape (Q factor > 45000, ER > 21 dB), a stable DC tuning efficiency (0.4 pm V^−1^), a high AC modulation speed (>3 GHz), and a high voltage output (*V*_pp_ > 300 V). Negligible performance degradations are observed after the system integration because the AlN modulator can inherit the high‐voltage from T‐TENG thanks to their capacitive nature. Toward practical applications, optical Morse code transmission and continuous human motion monitoring are demonstrated based on the two unique advantages respectively. This hybrid integration is a crucial demonstration toward future self‐sustainable wearable photonic ICs and tunable photonic sensors, which will find significant applications in IoT and human‐machine interface.

## 7 Experimental Section

##### Fabrication of the T‐TENG

The T‐TENG contains three layers: a positive charge generation layer, a negative charge generation layer, and a spacer in between. First, the conductive textile was cut into a size of 4 cm × 4 cm, which is made of metalized fabric (polyester Cu) coated with an adhesive. To fabricate the positive charge generation layer, a thin and wrinkled nitrile film was attached to one side of a conductive textile, with the other side attached with non‐conductive textile for electrical insulation. Another conductive textile was coated with silicone rubber on the one side and attached with a non‐conductive textile on the other side for insulation as well. The coating process included first dispensing required amounts of Parts A and B of the EcoFlex 00‐30 into a mixing container (1A:1B by weight), followed by mixing the blend thoroughly for 3 min, then pasting the mixed solution onto the conductive textile surface, and finally curing by 20‐min baking at 70 °C. A thin and porous sponge film was cut into strips with a width of 2 mm and length of 36 mm as spacers. Last, the silicone rubber coated textile was stitched to the nitrile coated textile with the sponge spacers placed on the edges of the square in between them.

##### Fabrication of the AlN Modulator

The AlN modulator fabrication started from a commercially available 8‐in Si wafer covered by a thin layer of SiO_2_. A 120 nm TiN layer as the bottom electrode and a 50 nm Si_3_N_4_ layer as the SiO_2_ etching stop layer were deposited and patterned. A 2 µm SiO_2_ layer was deposited as the bottom cladding and planarized for the following 400 nm AlN layer deposition. Next, a 200 nm SiO_2_ layer was deposited and patterned as the hard mask for AlN etching. After the waveguide patterns were transferred to the AlN layer, the waveguides were cladded by another 2 µm planarized SiO_2_. Contact holes were opened followed by a 2 µm Al layer deposition and patterning for bottom electrode contact and top electrode formation. Finally, for fiber butt coupling, deep trenches of more than 100 µm were formed before wafer dicing.

##### Characterization of the Standalone T‐TENG

The open‐circuit voltage and short‐circuit current of the T‐TENG were measured by a Keithley 6514 Electrometer, and then recorded by an oscilloscope (Agilent DSO‐X3034A) connected to it. The cyclic impact force on the T‐TENG was applied by a force gauge testing system (Mecmesin Multitest 2.5‐i Test System). For the voltage and power measurements, resistor loads with varying values were connected with the T‐TENG and voltage on resistor loads is also measured by Keithley 6514 Electrometer.

##### Characterization of the Standalone AlN Modulator

The resistance and capacitance of the AlN modulator were measured by a Keithley 6514 Electrometer. As for the optical characterization, the light was emitted from a tunable laser source covering 1520 to 1610 nm with a minimum tuning step of 0.1 pm (Keysight 81960A Tunable Laser). The light was guided through a single‐mode‐maintaining polarization controller, focused to the inversed tapered waveguide by a tapered fiber (OZ Optics, TSMJ‐3A‐1550‐9/125‐0.25‐18‐2.5‐14‐3‐AR), and finally directed to the AlN modulator. The electrical signal was applied to the AlN modulator through a GSG probe with a 100 µm pitch (MPI T26A GSG100). In DC tuning characterization, a tunable DC voltage supply (Agilent E3631A) was connected to the GSG probe after the voltage was amplified by 20× using a voltage amplifier (FLC Electronics A400DI). The optical output spectrum from the AlN modulator was measured by a power meter (Keysight 81636B Power Sensor) which was synchronized with the tunable laser source. In AC modulation characterization, a pulse pattern generator (0.05–12.5 GHz, Anritsu MP1763C) was connected to the GSG probe after the *V*_pp_ was amplified to 10 V. The modulated optical output was received by a high‐speed receiver (Discovery Semiconductor. Inc.) and converted to RF output which was then captured by a digital storage oscilloscope with a sampling rate of 80 GSa s^−1^ (Agilent Technologies DSO93004L).

##### Characterization of the Integrated Wearable NENS

The general characterization was the same as the characterization of the AlN modulator. The difference lies in three aspects. First, the GSG probe was connected to the output from the textile T‐TENG. Second, the electric signal converted from the optical transmission by the high‐speed receiver was captured by an oscilloscope (Agilent DSO‐X3034A). Third, in the demonstration of system applications, the applied force on T‐TENG was generated by human motions instead of the force gauge.

##### Study Participation

Prior to participation in the experiments, informed consent was obtained from the volunteer in all experiments.

## Conflict of Interest

The authors declare no conflict of interest.

## Supporting information

Supporting Information

Supplemental Video 1

Supplemental Video 2

Supplemental Video 3
