# Supplementary material for: Wearable Triboelectric/Aluminum Nitride Nano‐Energy‐Nano‐System with Self‐Sustainable Photonic Modulation and Continuous Force Sensing
Source: Adv Sci (Weinh). 2020 Jun 19;7(15):1903636. doi: 10.1002/advs.201903636 (PMC7404172; doi:10.1002/advs.201903636)
Supplement: Supplementary file 1 — Supporting Information [file ADVS-7-1903636-s001.pdf]

© 2020 Wiley-VCH GmbH

ADVANCED SCIENCE
Open Access

Supporting Information

for *Adv. Sci.*, DOI: 10.1002/advs.201903636

Wearable Triboelectric / Aluminum Nitride Nano-Energy-Nano-System (NENS) with Self-Sustainable Photonic Modulation and Continuous Force Sensing

*Bowei Dong, Qiongfeng Shi, Tianyiyi He, Shiyang Zhu, Zixuan Zhang, Zhongda Sun, Yiming Ma, Dim-Lee Kwong, Chengkuo Lee**

Copyright WILEY-VCH Verlag GmbH & Co. KGaA, 69469 Weinheim, Germany, 2020.

Supporting Information

Wearable Triboelectric / Aluminum Nitride Nano-Energy-Nano-System (NENS) with Self-Sustainable Photonic Modulation and Continuous Force Sensing

*Bowei Dong, Qiongfeng Shi, Tianyiyi He, Shiyang Zhu, Zixuan Zhang, Zhongda Sun, Yiming Ma, Dim-Lee Kwong, Chengkuo Lee**

Note S1. Open-circuit voltage waveforms of the T-TENG under different impact force magnitudes

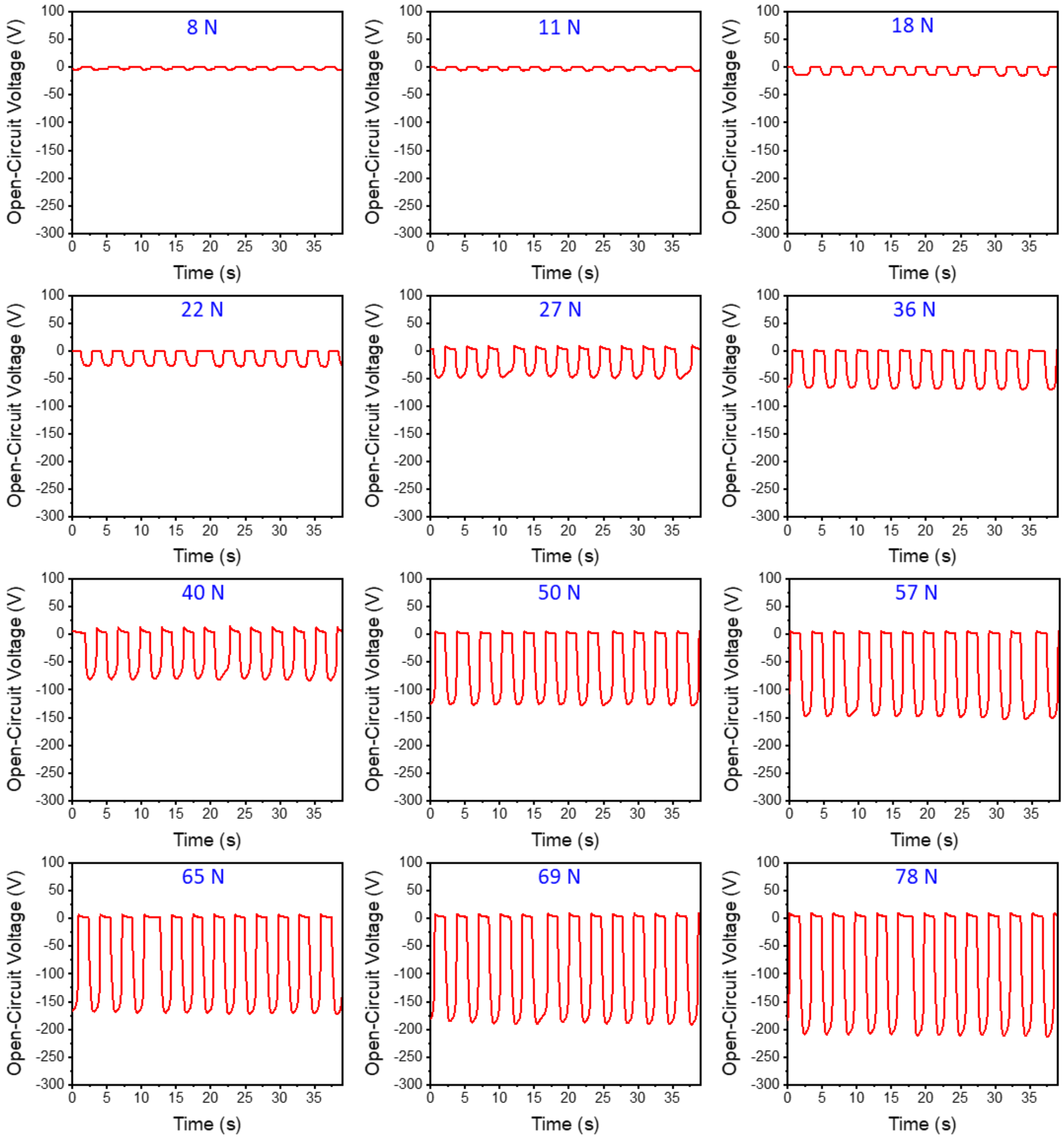

Figure S1. T-TENG open-circuit voltage under different applied periodic forces with discrete impact force magnitudes ranging from 8 N to 78 N. The speed of the load cell is 900 mm/min in all cases. The open-circuit voltage increases with the impact force magnitude in this range.

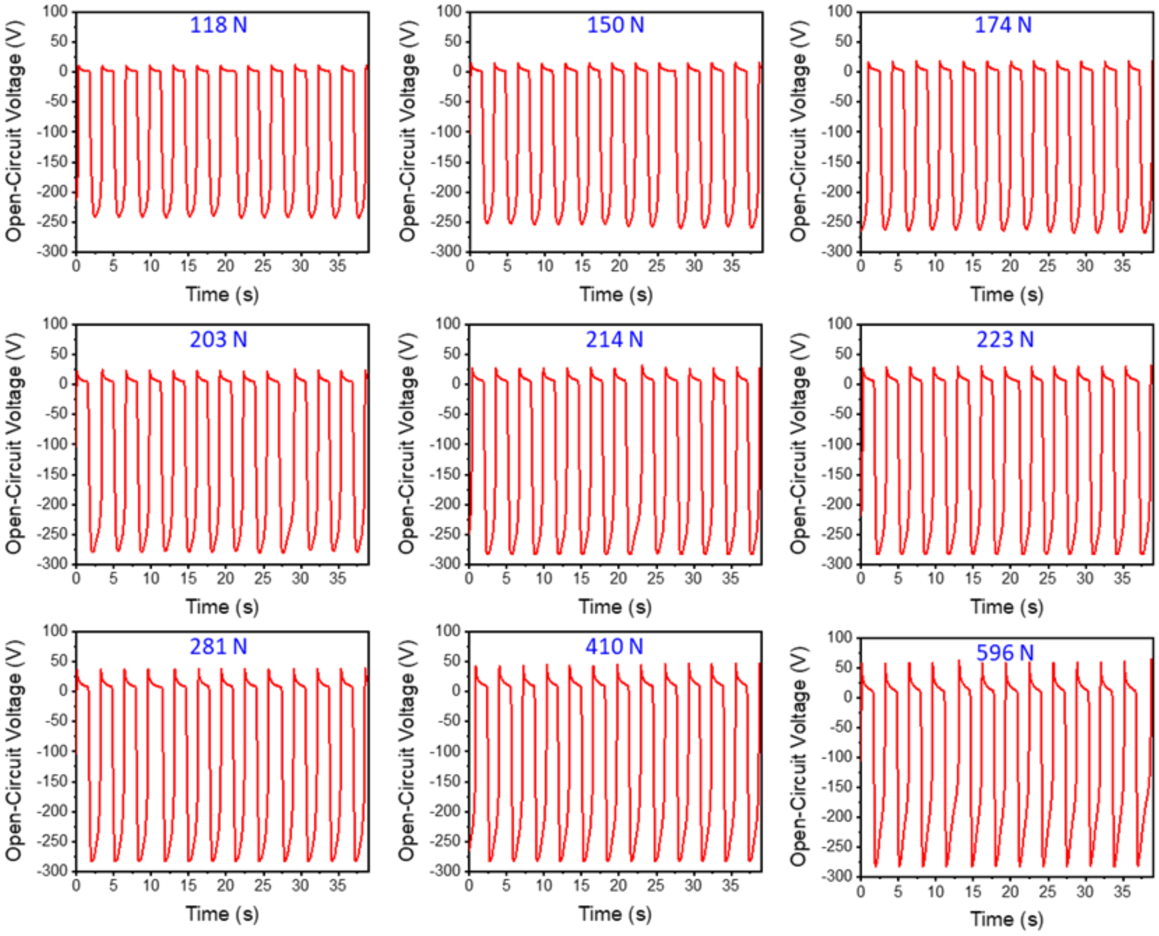

Figure S2. T-TENG open-circuit voltage under different applied periodic forces with discrete impact force magnitudes ranging from 118 N to 596 N. The speed of the load cell is 900 mm/min in all cases. The open-circuit voltage saturates when the impact force magnitude exceeds 200 N.

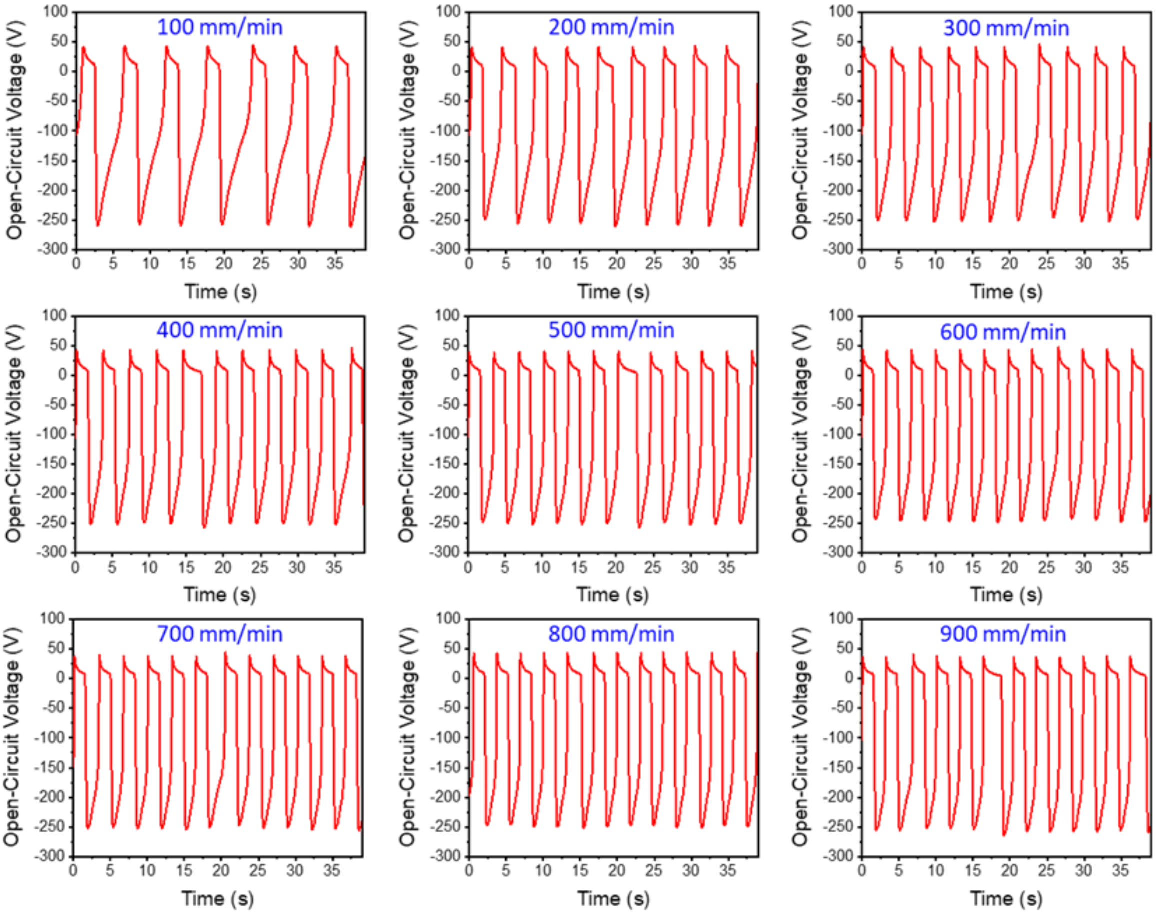

Figure S3. T-TENG open-circuit voltage under different periodic forces applied by the load cell moving at varying speeds ranging from 100 mm/min to 900 mm/min. The impact force magnitude is 200 N in all cases. The open-circuit voltage is not affected by different load cell speeds.

Note S2. Measuring the T-TENG output by finger tapping

The approach of measuring the T-TENG output by finger tapping is slightly different from other T-TENG output measurements. Tapping by a different number of fingers mainly affects the force and the contact area. Although the force can be easily monitored by a force sensor, the contact area is difficult to monitor. Therefore, on the surface of the T-TENG, we paste blocks with the area of 1 cm^2, 2 cm^2, 3 cm^2 to be compatible with the typical human fingertip area **(Figure S4(a))**. When the finger presses, the block presses the T-TENG with the pre-defined area so that the contact area is well controlled (see Figure S4(b) for a better illustration). The force sensor to which the T-TENG is attached reads the force while the T-TENG output is recorded by Keithley. In such a way, the three relevant physical quantities, namely the force, the T-TENG output, and the contact area are properly recorded.

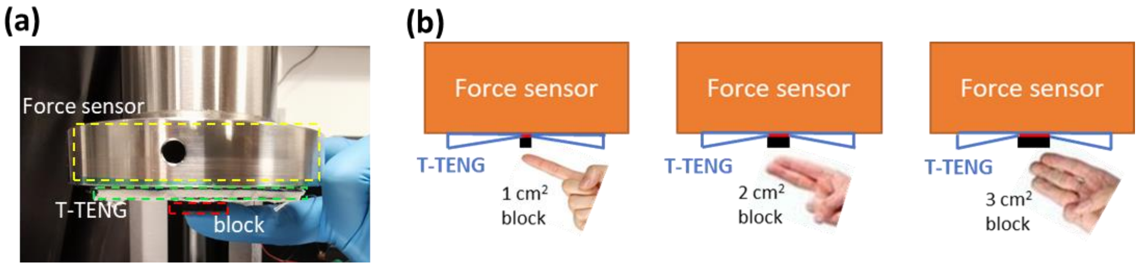

Figure S4. (a) Approach of measuring the T-TENG output by finger tapping. (b) Schematic illustration for the measurement of T-TENG output by finger tapping.

Note S3. Tailoring resonant wavelengths and resonant lineshapes

Table S1. The λ_R and FSR of AlN microring resonators with varying radius

Radius (μm)	30	40	50	60	80	100
λ_R (nm)	1554.442	1555.581	1556.268	1556.734	1557.413	1557.938
FSR (nm)	6.166	4.638	3.716	3.077	2.32	1.868

λ_R: resonant wavelength

FSR: free spectral range

Note S4. Theoretical analysis of the effect of fabrication variations on AlN modulator DC tuning efficiency

The circulating power in the MRR is given by:

$$P = \frac{\alpha^2(1-|t|^2)}{1+\alpha^2|t|^2-2\alpha|t|cos\theta}$$ Equation S1

where α is the loss coefficient of the ring, *t* is a coupler parameter depending on MRR design, and θ is the accumulated phase as the light travels for one circumference. On resonance, P is maximum so that $cos\theta = 1$. Thus, $\theta = 2\pi m$, where m is an integer. By expressing θ using the known physical parameters, the following relation is derived:

$$2\pi n_{eff}R = m\lambda$$ Equation S2

where n_{eff} is the effective index of the propagating mode and R is the ring radius. At a specific λ_R in an AlN MRR, both R and m are fixed. $\Delta\lambda$ only depends on Δn_{eff} which is solely determined by the E-field across the AlN MRR. Consequently, the tuning efficiency of the AlN MRR is independent of the device geometry variations.

Note S5. Deriving the r_{13} electro-optic coefficient of AlN

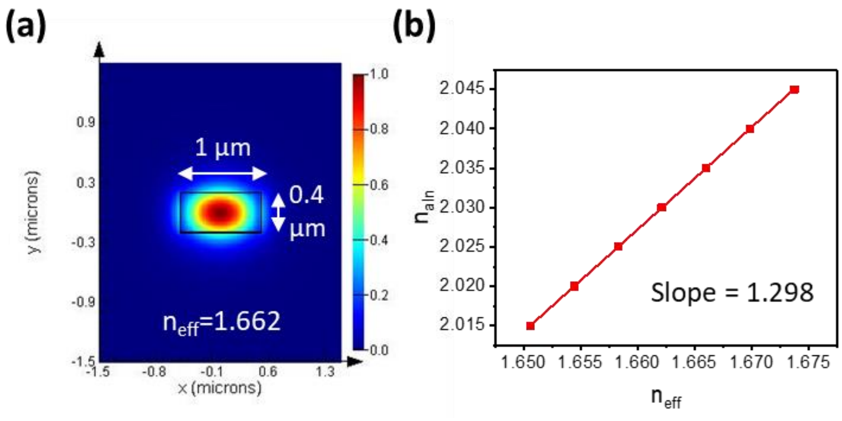

Figure S5. Simulation results to assist the derivation of the r_{13} electro-optic coefficient of AlN. (a) Mode profile of the fundamental mode in the AlN waveguide. (b) The sensitivity of the effective index of the propagating mode (n_{eff}) to the AlN material refractive index (n_{AlN}).

According to the finite-difference frequency-domain (FDFD) simulation with n_{AlN} = 2.03 at 1555 nm, n_{eff} is 1.662 as shown in **Figure S5(a)**. Thus, $\frac{2\pi R}{m} = \frac{\lambda}{n_{eff}} = 935.94\,nm$, so $d\lambda = 935.94 \times dn_{eff}$. From the experimental result of $\Delta\lambda = 160\,pm$ when 400 V is applied **(Figure 3(d))**, we derive $\Delta n_{eff} = 1.71 \times 10^{-4}$ at 400 V DC bias. This corresponds to 4.274 × 10^{-7} /V change rate in n_{eff} and 5.55 × 10^{-7} /V in n_{AlN} according to **Figure S5(b)**. The E-field crossing the AlN waveguide can be approximated by:

$$E = \frac{V}{t_{AlN} + \frac{(t_{upper} + t_{bottom}) \times \varepsilon_{AlN}}{\varepsilon_{SiO2}}} \qquad \text{Equation S3}$$

where t_{AlN} is the AlN waveguide thickness (400 nm), t_{upper} and t_{bottom} are the upper and bottom cladding thickness (both 2 µm), ε_{SiO2} and ε_{AlN} are the dielectric constant of SiO_2 (3.9) and AlN (~ 10). E is subsequently 3.68 × 10^{7} V/m.

Based on the relation between AlN's refractive index change and the applied E-field:

$$|\Delta n_{AlN}| = 0.5 r_{13} {n_{AlN}}^3 |E| \qquad \text{Equation S4}$$

AlN's r_{13} electro-optic coefficient of 1.44 pm/V is obtained.

Note S6. T-TENG output voltage reduction caused by the BNC cable connection

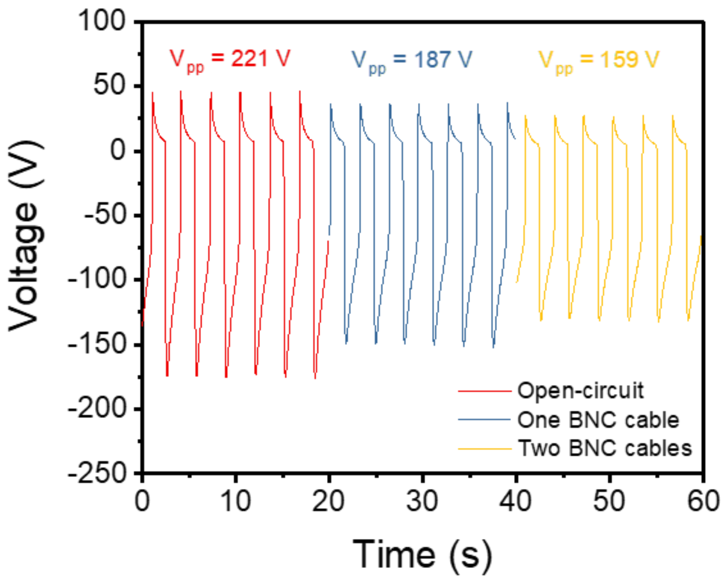

Figure S6. T-TENG output voltage reduction caused by the BNC cable connection.

After connecting the T-TENG module to the AlN modulator using two BNC cables, it is observed the maximum T-TENG output voltage drops from 350 V to 235 V (67.14% remaining) at 600 N force. To understand the reason for the voltage reduction, at 200 N force we test the TENG output voltage when it is connected to the BNC cables but isolated from the AlN modulator. The measurement result is presented in **Figure S6**, the peak to peak voltage V_{pp} firstly decreases from 221 V to 187 V (84.6% remaining) when one BNC cable is added. Then V_{pp} further decreases to 159 V (71.9% remaining) when two BNC cables are added. This suggests that the voltage reduction is mainly caused by the BNC cables instead of the AlN modulator with minuscule capacitance.

Note S7. Twenty-one optical transmission waveforms at different operation wavelengths

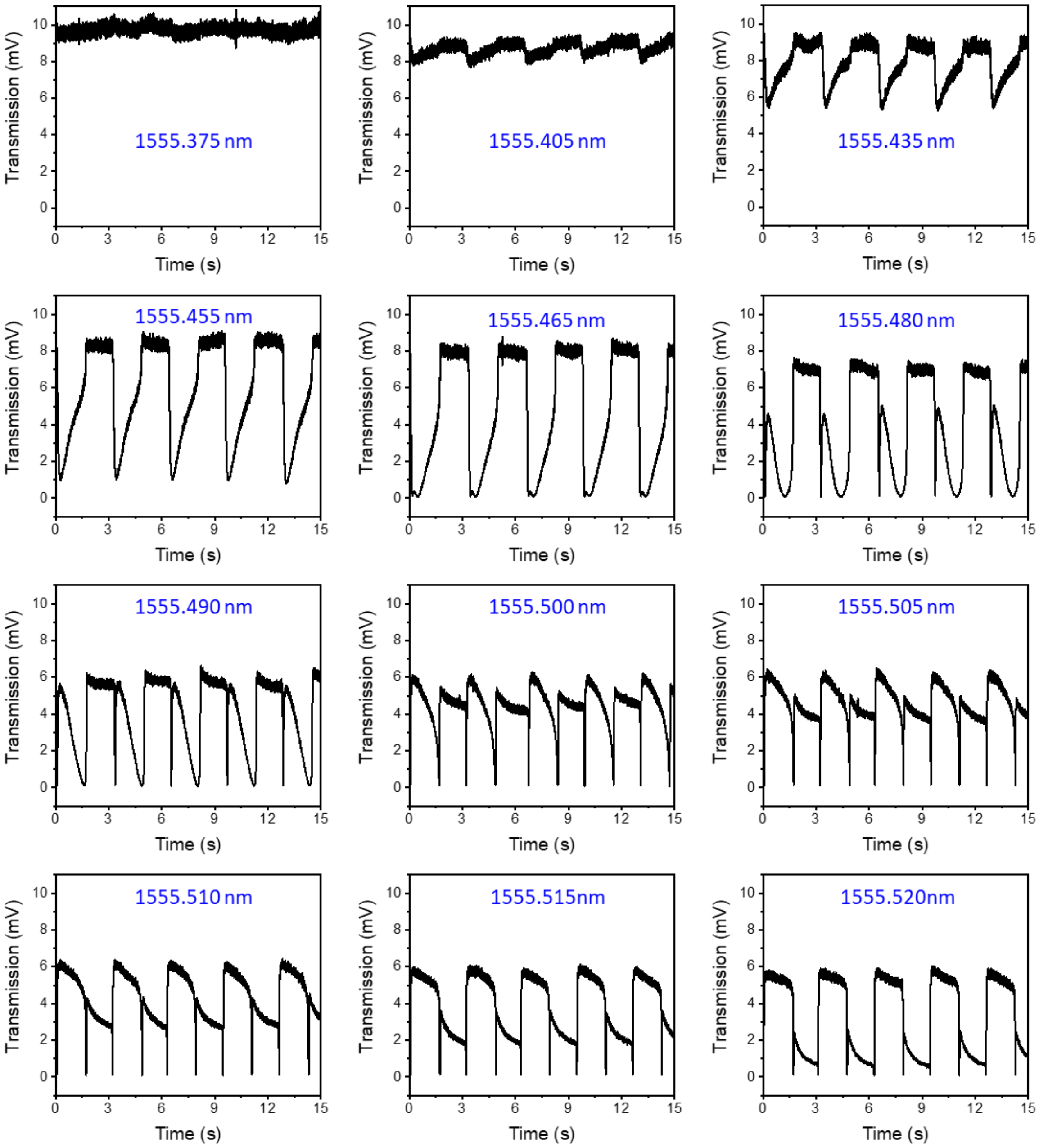

Figure S7. The optical transmission waveforms at discrete operation wavelengths ranging from 1555.375 nm to 1555.520 nm. The applied TENG output voltage waveform is the same as **Figure 4(d)**.

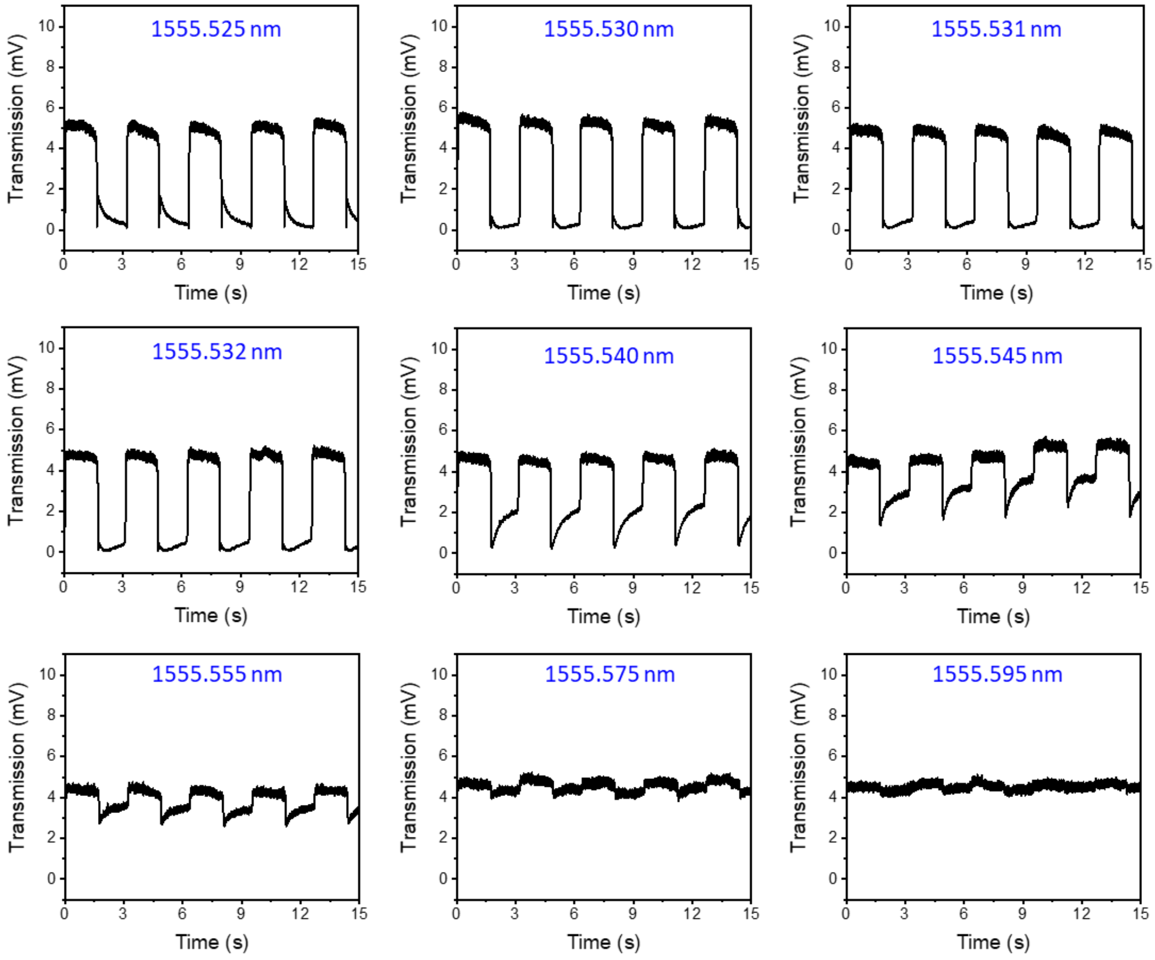

Figure S8. The optical transmission waveforms at discrete operation wavelengths ranging from 1555.525 nm to 1555.595 nm. The applied TENG output voltage waveform is the same as **Figure 4(d)**.

Note S8. The calibration curve for converting the optical transmission to the impact force magnitude

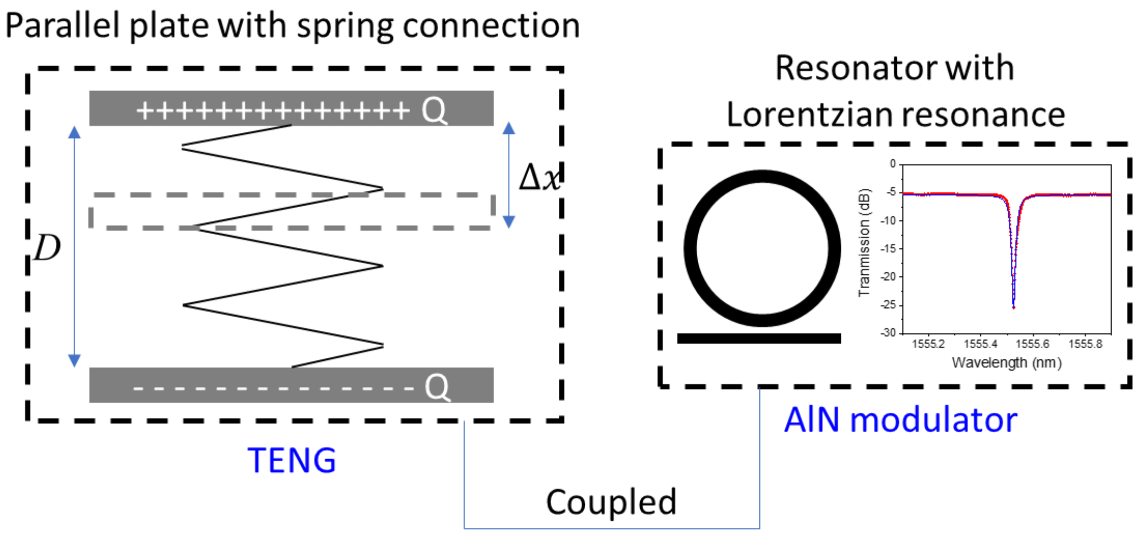

Figure S9. The physical model proposed for developing the optical transmission vs. the impact force magnitude calibration curve.

As shown in **Figure S9**, the T-TENG is considered as a parallel plate capacitor where the two plates with constant charge +Q and -Q are connected by a spring. Mechanically, the T-TENG under applied force can be described by the stress-strain curve:

$$F = K(\Delta x)^N \qquad \text{Equation S5}$$

where F is the impact force magnitude, Δx is the displacement of the spring, K is the strength coefficient, and N is a measure of material work hardening behavior.

Electrically, the voltage V across the parallel plate capacitor is described by the parallel plate capacity equation:

$$V = \frac{Q(D-\Delta x)}{\epsilon A} \qquad \text{Equation S6}$$

where D is the original separation of the two parallel plates, ϵ is the permittivity, and A is the area of parallel plates.

According to the DC tuning efficiency result as shown in **Figure 3(c)**, the resonant wavelength shift is given by:

$$\varDelta\lambda = 4 \times 10^{-4} \times V \qquad \text{Equation S7}$$

where $\varDelta\lambda$ is the resonant wavelength shift (in unit of nm) in the AlN MRR caused by the applied voltage.

The transmission T from the AlN MRR is described by the Lorentzian function as shown in **Figure 3(b)**:

$$T = T_0 + \frac{2B}{\pi} \times \frac{\omega}{4\varDelta\lambda^2 + \omega^2} \qquad \text{Equation S8}$$

where T_0 and B are constants, ω is the 3-dB bandwidth of the Lorentzian function. ω is 3.4×10^{-2} nm according to the fitting result in **Figure 3(b)**.

From **Figure 5(a)** to **Figure 5(c)**, we have the following boundary conditions:

$$V = -120\ \text{V}, \text{when } F = 0 \qquad \text{Boundary Condition SB1}$$

$$T = 0\ \text{mV}, \text{when } V = -120\ \text{V so } \varDelta\lambda = 4 \times 10^{-4} \times 120 = 4.8 \times 10^{-2}\text{nm}$$

Boundary Condition SB2

By substitute Equation S5 into S6 and apply the boundary condition B1, we obtained

$$\frac{Q}{\epsilon A} = -\frac{120}{D} \qquad \text{Equation S9}$$

By applying the boundary condition B2 on Equation S8, we obtained

$$T_0 = -\frac{2B}{\pi} \times \frac{\omega}{4\times(4.8\times10^{-2})^2 + \omega^2} \qquad \text{Equation S10}$$

Therefore, combining Equation S5 to Equation S10, we obtained:

$$T = \frac{2B}{\pi} \times 3.4 \times 10^{-2} \times [-\frac{1}{1.0372\times10^{-2}} + \frac{1}{1.156\times10^{-3} + 6.4\times10^{-7}\times\left[\frac{120}{D}\times\left(D-\left(\frac{F}{K}\right)^{\frac{1}{N}}\right)\right]^2}$$

Equation S11

Note S9. Direct comparison between the signal generated in the TENG+photonics+MCU system and the signal generated in the TENG+MCU system for the Morse code transmission and continuous human motion monitoring applications

For the Morse code transmission, it is a method used in telecommunication to encode text characters as standardized sequences of two different signal durations, called dots and dashes. If the TENG is directly connected to the MCU, the signal duration cannot be well controlled since only two output pulses can be generated in one TENG contact-separation cycle. As shown in Figure S10, we make a direct comparison between the signal generated in the TENG+photonics+MCU system and the signal generated in the TENG+MCU system. In the TENG+photonics+MCU system, the TENG output voltage as well as the optical transmission can be maintained for a long duration (Figure S10(a)). On the contrary, in the TENG+MCU system, only voltage pulses can be obtained so that the signal duration cannot be long (Figure S10(b)). The corresponding mechanisms are further illustrated in Figure S10(c) and Figure S10(d). In the TENG+photonics+MCU system, since no electrical charges flow in the open-circuit condition, the TENG acts as a parallel-plate capacitor once the surface charges are generated. The voltage depends on the separation distance between the two TENG surfaces (or, the gap of the capacitor). Therefore, as long as the separation distance is fixed, the TENG voltage is stable and can last for a long duration. In the TENG+MCU system, the induced surface charges can drive charges in the electrodes flow through the load resistance of the MCU. When the separation distance changes, the induced charges flow to make the system in the equilibrium state in a short time duration. Then the voltmeter reads 'zero' and provides no information since no current flows through the load resistance.

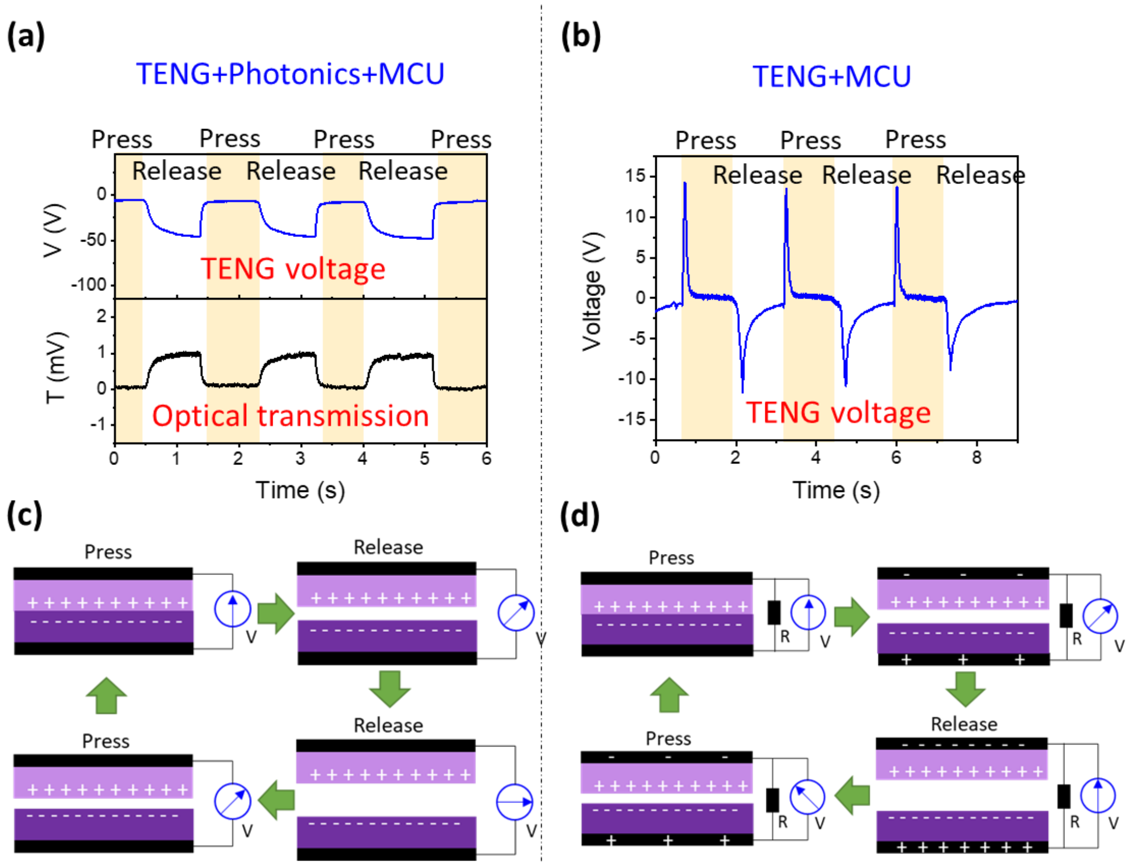

Figure S10. For Morse code transmission, a direct comparison between the signal generated in (a) the TENG+photonics+MCU system and (b) the signal generated in the TENG+MCU system, as well as (c)&(d) the corresponding mechanisms.

For the human motion monitoring, directly connecting the TENG to the MCU cannot realize continuous motion monitoring. As shown in Figure S11, in the arm patting demonstration, only pulses are seen in the TENG+MCU system while the TENG+photonics+MCU system can continuously monitor the human motion (Figure S11(a)). The reason is the same as discussed above for the Morse code transmission. Consequently, if we would like to zoom in to one arm patting cycle to read more information, the TENG+photonics+MCU system provides complete information (Figure S11(b)). In the first 0.1 seconds, the hand pats the arm quickly so a fast rise in the optical transmission is seen. In the next 0.2 seconds, the hand

presses the arm slowly and leads to a slower increase in the optical transmission. Nonetheless, in the TENG+MCU system (Figure S11(c)), the voltage rises fast due to the current flow in the MCU load resistance when the hand pats the arm. Then the voltage drops since the system reaches the equilibrium state where no current flows, leading to the loss of information in the next 0.2 seconds.

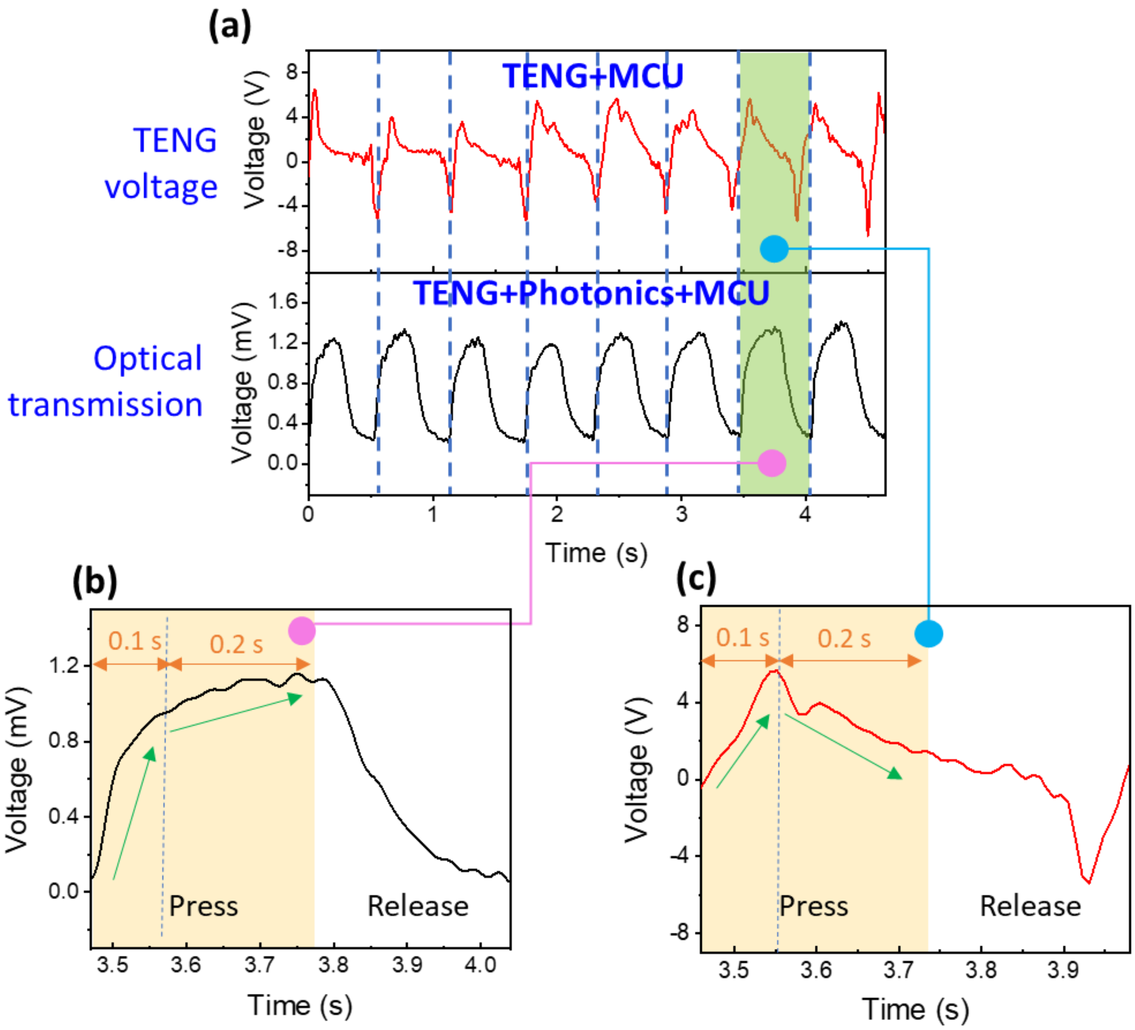

Figure S11. For human motion monitoring, (a) a direct comparison between the detected signal in the MCU module of the TENG+photonics+MCU system and the TENG+MCU system. (b)&(c) Zoom-ins to the 7th arm patting cycle.

In summary, directly connecting the TENG to the MCU cannot achieve a stable TENG voltage as well as the continuous monitoring applications. Adding a capacitor-like AlN photonics modulator helps to address this issue by decoupling the electrical triggering signals and the optical readout signals so that the TENG open-circuit voltage can be read by an MCU in a very simple manner to benefit continuous motion monitoring and Morse code transmission which needs a relatively long stable signal duration.

Supporting Videos

Video S1. Dynamic self-sustainable optical Morse code transmission of 'HINUS'. The top left panel shows the dynamic optical signal. The bottom left panel shows the pre-measured Morse code reference. The bottom middle panel shows the software window/interface. The right panel shows the real-life human control. The self-sustainably modulated optical signals are received by a photodetector and converted to electric signals. The electric signals are processed by a microcontroller unit (MCU) and translated to preset alphabets by a home-built software. The "bar" and "dot" in Morse code definition is achieved by the long and short duration of the T-TENG voltage output. The data transmission conveying the message of "HINUS" is successfully demonstrated.

Video S2. Continuous human motion monitoring – arm patting. The wearable NENS is pasted on a human arm. Both the T-TENG voltage output and the modulated optical signals are presented in the oscilloscope. The optical transmission waveform can reproduce the T-TENG voltage output which is determined by the force exerted on the wearable NENS. Arm patting and pressing can be continuously monitored.

Video S3. Continuous human motion monitoring – walking. The wearable NENS is pasted on a human sole. Both the T-TENG voltage output and the modulated optical signals are presented in the oscilloscope. The optical transmission waveform can reproduce the T-TENG voltage output which is determined by the force exerted on the wearable NENS. Walking patterns can be continuously monitored.
